# Structure-based evidence for the enhanced transmissibility of the dominant SARS-CoV-2 B.1.1.7 variant (Alpha)

**DOI:** 10.1038/s41421-021-00349-z

**Published:** 2021-11-09

**Authors:** Shuai Xia, Zuoling Wen, Lijue Wang, Qiaoshuai Lan, Fanke Jiao, Linhua Tai, Qian Wang, Fei Sun, Shibo Jiang, Lu Lu, Yun Zhu

**Affiliations:** 1 Key Laboratory of Medical Molecular Virology (MOE/NHC/CAMS), School of Basic Medical Sciences and Biosafety Level 3 Laboratory, Shanghai Institute of Infectious Disease and Biosecurity, Fudan University, Shanghai, China; 2 National Key Laboratory of Biomacromolecules, CAS Center for Excellence in Biomacromolecules, Institute of Biophysics, Chinese Academy of Sciences, Beijing, China; 3 University of Chinese Academy of Sciences, Beijing, China; 4 Bioland Laboratory (Guangzhou Regenerative Medicine and Health Guangdong Laboratory), Guangzhou, Guangdong China

**Keywords:** Structural biology, Molecular biology

Dear Editor,

Severe acute respiratory syndrome coronavirus 2 (SARS-CoV-2), the causative agent of coronavirus disease 2019 (COVID-19), has resulted in over 245 million infections and ~5 million deaths, severely threatening global public health. Moreover, numerous SARS-CoV-2 variants of concern (VOCs) with even higher transmissibility, such as B.1.1.7 (Alpha), B.1.351 (Beta), B.1. 617.2 (Delta), and C.37 (Lambda), are continuously emerging^1^. Monitoring these dominant SARS-CoV-2 variants and exploring the potential reason for their higher transmissibility are important for controlling the current COVID-19 pandemic. B.1.1.7, the first SARS-CoV-2 VOC, was first identified on September 20, 2020 in the United Kingdom (UK) and quickly became the locally dominant circulating mutant. Currently, it has spread to more than 90 countries, causing ~10 million infections (https://cov-lineages.org/global_report.html). Previous studies have reported that the B.1.1.7 variant shows a significant increase in the effective reproductive rate with increased secondary attack rate^2^. However, basic studies elucidating the mechanism underlying the increased infectivity of the B.1.1.7 variant are lacking. In particular, structural studies of the complex containing the B.1.1.7 mutant spike (S) protein and hACE2 receptor are not currently available. Therefore, it is still unclear whether the higher infectivity of the full-length B.1.1.7 mutant S protein is related to its increased receptor-binding affinity.

The rapid spread of B.1.1.7 has increased concern about those natural mutations in the S protein, including 69–70 deletion, 144 deletion, N501Y, A570D, D614G, P681H, T716I, S982A, and D1118H (Fig. 1a). Previous studies reported that the B.1.1.7 mutant S protein showed greater receptor-binding affinity than the wild-type (WT) S protein^3^. In contrast, some researchers found a reduced binding affinity between B.1.1.7 and hACE2^4,5^. These conflicting results challenge the assumption of “the higher infectivity of B.1.1.7 mediated by the enhanced receptor-binding affinity”. Therefore, it is necessary to reveal the structure of the B.1.1.7 S-hACE2 complex to fully understand the potential mechanism underlying the high infectivity of the B.1.1.7 variant.

**Fig. 1:**
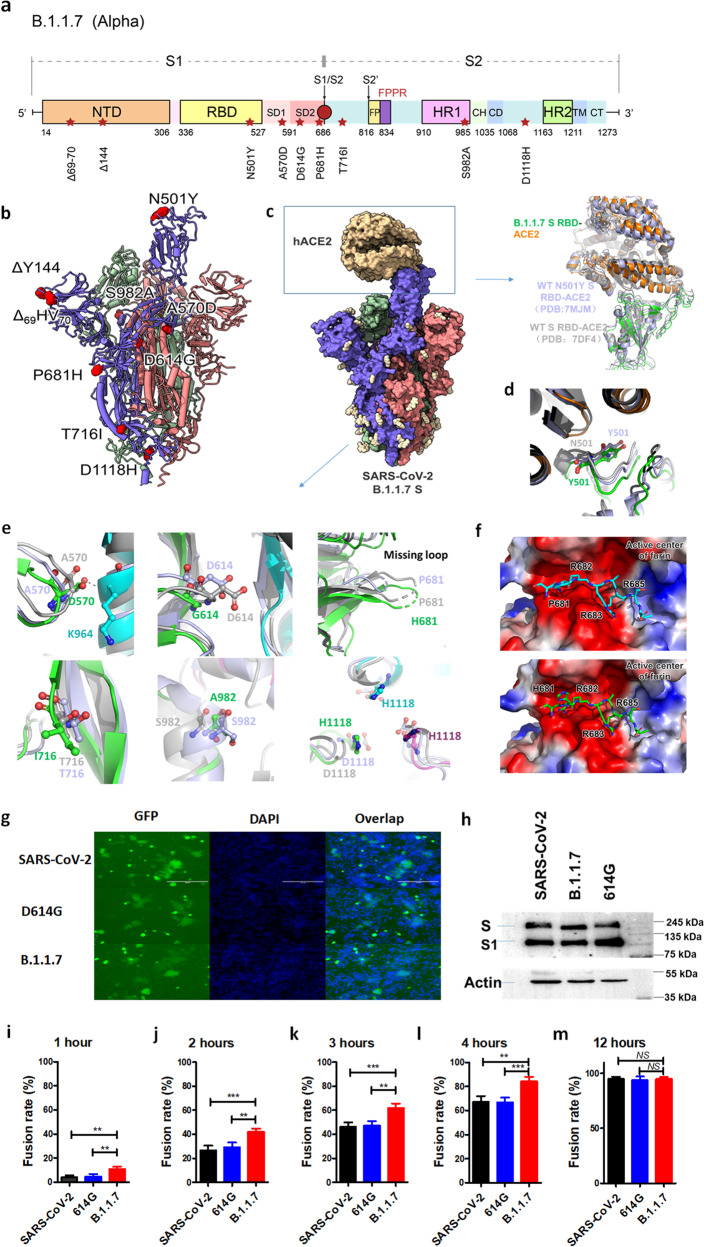
Structural and functional effects of mutations in the S protein of the SARS-CoV-2 B.1.1.7 (Alpha) variant on human lung cells. **a** Schematic representation of the B.1.1.7 SARS-CoV-2 S protein with ten natural mutations. The S1 subunit contains mainly an N-terminal domain (NTD), a receptor-binding domain (RBD), and subdomains 1 and 2 (SD1 and SD2). The S2 subunit contains a fusion peptide (FP), fusion peptide proximal region (FPPR), heptad repeats 1 and 2 (HR1 and HR2), a central helix (CH), a connector domain (CD), a transmembrane domain (TM) and a cytoplasmic tail (CT). **b** Overall structure of SARS-CoV-2 B.1.1.7 S in the 1-RBD-up state, indicating the natural mutations. **c** Overall structure of the SARS-CoV-2 B.1.1.7 S-ACE2 complex. The interface between the RBD and ACE2 is magnified to compare the structures of WT S-ACE2 (gray) and N501Y S-ACE2 (light blue). **d** The N501Y mutation site in the three structures mentioned in (**c**). **e** The A570D, D614G, P681H, T716I, S982A and D1118H mutation sites in the three structures mentioned in (**c**). **f** Structural model of furin with the cleavage sites in the SARS-CoV-2 and B.1.1.7 variants. **g** Representative images of cell–cell fusion between 293T/SARS-CoV-2(WT)/EGFP, 293T/SARS-CoV-2(D614G)/EGFP, 293T/SARS-CoV-2(B.1.1.7)/EGFP effector cells and target cells (Calu-3) after coculture for 6 h. Scale bars, 400 µm. **h** Western blot analysis of S protein expression in effector cells. **i**–**m** Statistical analysis of fusion rates mediated by the WT, D614G, and B.1.1.7 S protein after coculture for 1 h (**i**), 2 h (**j**), 3 h (**k**), 4 h (**l**), and 12 h (**m**). Asterisks indicate significant differences. *P < 0.05, **P < 0.01, ***P < 0.001. *NS*, not significant.

To determine the structural basis for the enhanced infectivity mediated by the S protein of the dominant SARS-CoV-2 B.1.1.7 variant, we used cryo-electron microscopy (cryo-EM) single-particle analysis (SPA) to solve the structures of B1.1.7 S in the 1-RBD-up state and the B1.1.7 S-hACE2 complex (Supplementary Fig. S1 and Table S1) with resolutions of 3.7 and 4.1 Å, respectively, according to the gold standard Fourier shell correlation (FSC) coefficient of 0.143 (Supplementary Fig. S2). To improve protein stability for cryo-EM data processing, proline substitutions at K986 and V987 and a “GSAS” substitution at the furin cleavage site (S1/S2 site, R682 to R685) were introduced into the purified S protein, according to a previous report^6^. In the two structures, the most stable region with the highest local resolution is the helical bundle of the S2 subunit (Supplementary Fig. S2 and Video S1), consistent with the previous reports^3,6^. In the S-ACE2 complex structure, the interface between the S RBD and human ACE2 is clearly discernible (Supplementary Video S2), suggesting that the two proteins form a stable binding state in the absence of any crosslinker^7^. Ten point mutations were identified in the S protein of SARS-CoV-2 B.1.1.7 compared to the WT S protein (Fig. 1b). They are scattered in different locations throughout the trimeric structure in the prefusion state (Fig. 1b and Supplementary Fig. S3). Among the mutations, the _69_HV_70_ and Y144 deletions in the N-terminal domain (NTD), as well as the P681H mutation near the furin cleavage site, could not be traced in the cryo-EM map because they are located in the flexible loop region. The remaining six sites could be identified in both the S and S-ACE2 structures.

Despite these deletions and mutations, the overall structure of SARS-CoV-2 B.1.1.7 S changed little compared with that of WT S (Supplementary Figs. S3 and S4). Regarding the trimeric S protein in the 1-RBD-up state, our model is highly conserved with respect to the reported structures of the WT virus (EMD-21457, PDB entry 6VYB) and B.1.1.7 variant (EMD-23558, PDB entry 7LWV), with root-mean-square deviation (RMSD) values of 1.2 Å and 1.1 Å, respectively (Supplementary Fig. S4). Regarding the S-ACE2 complex structure, our model is also similar to that of the SARS-CoV-2 S-ACE2 complex (EMD-30661, PDB entry 7DF4) and the N501Y-mutated S-ACE2 complex (EMD-23878, PDB entry 7MJM), with RMSD values of 1.4 and 1.2 Å, respectively^3^ (Supplementary Fig. S4). These results suggest that the natural substitutions in the B.1.1.7 S protein affect neither its overall architecture nor its recognition of and binding to hACE2 (Fig. 1c). It was reported previously that the ACE2 binding will alter the position of open RBD in WT SARS-CoV-2 by a rigid-body rotation away from the trimer axis^8^. Our complex structure suggested that the B.1.1.7 S may maintain a more favorable conformation in advance before hACE2 engagement.

However, in some localized regions, we found evidence suggesting that these residue substitutions may promote enhanced viral fusion activity and, hence, increased infectivity. For example, similar to a previous study^3^, the Y501 residue could provide more interactions than N501 through π-π stacking or hydrogen bonding. Despite this possibility, few changes were found at the RBD-hACE2 interface in our complex structure (Fig. 1d). However, we did find some clues for the other mutation sites. For the A570 in the C-terminal domain (CTD) of the S1 subunit, the D570 substitution generates a new hydrogen bond to K964 in the S2 subunit of the other protomer, improving the overall stability of the S-trimer (Fig. 1e). In addition, the G614 residue does not participate in the original interactions of D614 with other residues (Fig. 1e), such as the D614-K854 salt bridge, which may help to prevent premature dissociation of the G614 trimer, as recently reported^9^. Similarly, the T716I and S982A substitutions abrogate the original interactions of T716 and S982, respectively, with the related residues, suggesting local destabilizing effects (Fig. 1e) for increasing the propensity for the “up” RBD conformation^10^. Finally, the D1118H substitution forms a symmetric histidine triad that may help stabilize the S-trimer through water-mediated interactions (Fig. 1e). Consistently, compared to the WT SARS-CoV-2 pseudovirion, T716I, and S982A pseudovirions showed higher thermostability, whereas A570D and D1118H pseudovirions showed metastable as well as D614G or B.1.1.7 pseudovirion (Supplementary Fig. S5).

Although the P681H substitution located in the flexible loop could not be traced in the cryo-EM map (Fig. 1e), we speculated its role based on the structure and function of furin. The crystal structure of furin with its inhibitor reveals its negatively charged substrate-binding pocket and explains its stringent requirement for arginine-rich substrates (Supplementary Fig. S6)^11^. Based on this structure, we modeled the complex structure of furin with its cleavage site in the S proteins of the original SARS-CoV-2 and the B.1.1.7 variant (Fig. 1f). We found that the cleavage sequence of _682_RRAR_685_ docks well into the substrate-binding pocket of furin through charge complementation and space matching, while P681 seems to contribute little to this interaction. Substitution of histidine for proline may further increase the binding affinity between the substrate and enzyme, thus promoting furin cleavage activity.

SARS-CoV-2 infects the human body mainly through its binding to, fusion with, and entry into epithelial cells in the human airway. Thus, we hypothesized that the B.1.1.7 S protein might mediate enhanced viral fusion and entry into lung cells that naturally and highly express the hACE2 receptor and numerous proteases, such as TMPRSS2 and TMPRSS11a, which can directly mediate viral fusion and entry at the cell surface^12^. Therefore, we used human lung-derived cell line Calu-3 as target cells to assess the putative increased transmissibility of the B.1.1.7 variant commencing with its S-mediated membrane fusion process. As shown in Fig. 1g, h, effector cells (293T/S/GFP) bearing B.1.1.7 S protein fused with Calu-3 cells with an efficacy equal to that of effector cells expressing the equal amounts of WT or D614G mutant S protein after co-incubation for 8 h. Nevertheless, we found different kinetics mediated by those S proteins. At early time points, including 1, 1.5, 2, 3, 4, and 6 h of coculture, the B.1.1.7 S protein mediated a higher fusion efficiency than the WT or D614G S protein (Fig. 1i–m and Supplementary Fig. S7). However, no appreciable difference in fusion kinetics between the WT, D614G or B.1.1.7 S protein was observed in 293T/ACE2 or 293T/ACE2/TMPRSS2 cells, consistent with previously reported^13^ (Supplementary Figs. S8 and S9), probably owing to the limited sensitivity of the target cells. It has been shown that the hACE2-TMPRSS2 complex and tyrosine protein kinase receptor UFO (AXL) are widely distributed in human lung cells and benefit viral infection^12,14^. Indeed, we also found that human lung cells, combined with cell–cell fusion kinetics, were sensitive enough for exploitation by the propensity of the B.1.1.7 variant toward increased infectivity, making epithelial lung cells an ideal model to evaluate membrane fusion and functional alterations of other emerging SARS-CoV-2 variants. In addition, serum from mice vaccinated with the WT SARS-CoV-2 RBD exhibited comparable inhibitory potency against the cell–cell fusion process and pseudovirus infection mediated by the WT, D614G or B.1.1.7 S proteins (Supplementary Fig. S10), suggesting that B.1.1.7 can still be equally restricted by the current SARS-CoV-2 vaccines.

Overall, in this study, we found that the mutations in the B.1.1.7 S protein can significantly enhance its viral fusion activity and its infectivity. Based on the structure of the B.1.1.7 S-trimer/hACE2 complex, we revealed multiple structural effects of those mutations on the S protein, such as enhancing ACE2 affinity, enhancing the prefusion state stability, increasing the propensity for the RBD “up” conformation, and promoting furin cleavage activity, all of which might effectively increase viral fusion activity and infectivity in the human airway environment. Therefore, these findings may serve as a guide for monitoring current and future SARS-CoV-2 VOCs.

## Supplementary information

Supplementary Information

Supplementary Video S1

Supplementary Video S2
