# Supplementary material for: Structure-based evidence for the enhanced transmissibility of the dominant SARS-CoV-2 B.1.1.7 variant (Alpha)
Source: Cell Discov. 2021 Nov 9;7:109. doi: 10.1038/s41421-021-00349-z (PMC8576028; doi:10.1038/s41421-021-00349-z)
Supplement: Supplementary file 1 — Supplementary Information [file 41421_2021_349_MOESM1_ESM.pdf]

Structure-based evidence for the enhanced transmissibility of the dominant SARS-CoV-2 B.1.1.7 variant (Alpha)

Shuai Xia[1]*, Zuoling Wen[2,3]*, Lijue Wang[1]*, Qiaoshuai Lan[1], Fanke Jiao[1], Linhua Tai[2,3], Qian Wang[1], Fei Sun[2, 3,4], Shibo Jiang[1]†, Lu Lu[1]†, Yun Zhu[2]†

Affiliations:

[1]Key Laboratory of Medical Molecular Virology (MOE/NHC/CAMS), School of Basic Medical Sciences and Biosafety Level 3 Laboratory, Shanghai Institute of Infectious Disease and Biosecurity, Fudan University, Shanghai 200032, China.

[2] National Key Laboratory of Biomacromolecules, CAS Center for Excellence in Biomacromolecules, Institute of Biophysics, Chinese Academy of Sciences, Beijing 100101, China.

[3] University of Chinese Academy of Sciences, Beijing 100049, China.

[4] Bioland Laboratory (Guangzhou Regenerative Medicine and Health Guangdong Laboratory), Guangzhou 510005, Guangdong Province, China.

*These authors contributed equally to this work.

†Corresponding author. Email: zhuyun@ibp.ac.cn (Y.Z.); lul@fudan.edu.cn (L.L.); shibojiang@fudan.edu.cn(S.J.);

Methods:

Cells，protein and plasmids

The 293T cell line was obtained from ATCC (Manassas, VA, USA); the Calu-3 cell line was from the Chinese Academy of Science Cell Bank (Shanghai, China). 293T/ACE2 cells were kindly provided by L.D. All cell lines were cultured in Dulbecco’s Modified Eagle’s Medium (DMEM) with 10% fetal bovine serum (FBS). Human ACE2 protein (Q9BYF1, residues 18-740) with a 6His tag at the C terminal was obtained from Novoprotein Company (cat.: C419). Plasmids, including pAAV-SARS-CoV-2-S-IRES-EGFP, pAAV-SARS-CoV-2-S-D614G-IRES-EGFP, pAAV-SARS-CoV-2-S-B.1.1.7-IRES-EGFP, pC-DNA-3.1-SARS-CoV-2-S, pC-DNA-3.1-SARS-CoV-2-S-D614G, pC-SARS-CoV-2-S-B.1.1.7, pNL4-3.Luc.R-E and pAAV-IRES-EGFP, were synthesized or preserved in our laboratory.

Western blot

Western blot was performed using an anti-SARS-COV-2 S1 antibody and an anti-actin antibody. Briefly, after transfection for 36 hours, effector cells bearing S protein on their surface were collected. Samples were prepared to run an SDS-PAGE in 10% gels (Bio-Rad, Hercules, CA) and then transferred onto PVDF membranes. Membranes were blocked with 5% BSA in PBST for 2 hours, followed by incubation with a SARS-CoV-2 S1 antibody (Sino Biological Inc., Beijing, China, Cat: 40592-T62), or a beta-actin mouse McAb (ProteinTech, Manchester, UK, Cat：66009-1-Ig), for another 2 hours at room temperature. Horseradish peroxidase (HRP)-conjugated polyclonal Goat

anti-Rabbit IgG (1:5000) (DAKO, Cat：P0448) and Goat anti-Mouse IgG (1:5000) (Abcam, Cambridge, UK, Cat: ab6789) were used as secondary antibodies. Proteins were visualized using one-step ECL substrates (Meilunbio, Dalian, China).

Cell-cell fusion assays

Plasmid pAAV-IRES-S-EGFP encoding S protein and EGFP were transfected into 293T effector cells (293T/S/GFP). Huh-7 cells, naturally expressing human ACE2 receptors on the membrane surface, were used as target cells. 293T cells, transfected with plasmid pAAV-IRES-EGFP (293T/EGFP), were used as negative control. The effector cells (293T/S/GFP) were collected and resuspended. The free effector cells were added into target cells (Calu-3 cells) for coincubation for indicated time at 37 °C and then observed under the fluorescence microscope.

Pseudovirus infection assays

Pseudoviruses were produced by cotransfecting plasmids carrying wild-type or mutant SARS-CoV-2 S protein with pNL4-3.Luc.R−E− as described previously[1]. Then pseudoviruses in the supernatant were collected 60 h after transfection and frozen at −80 °C. After quantification with P24-ELISA, pseudoviruses were added into the target cells (293T/ACE2). Medium was changed at 12 h post-infection. After an additional 48 h, luciferase activity was tested by the Luciferase Assay System (Promega, Madison, WI, USA).

Protein expression and purification

B.1.1.7 SARS-CoV-2 S protein (residues 15-1205) contains a “RRAR” (residues

682-685) to “GSAS” substitution to abolish the furin cleavage site, a “KV” (residues 986-987) to “PP” substitution to stabilize the S protein in prefusion state, and a foldon trimerization motif at the C terminus, which was expressed and purified by Novoprotein Company (Shanghai, China).

Cryo-EM grid preparation and data acquisition

Purified SARS-CoV-2 S protein was incubated with purified human ACE2 protein (in molar ratio of 1:1.5) at room temperature for 1 hour. The mixture was then applied to a Superdex 200 column in PBS, and the elution peak corresponding to S-ACE2 complex was collected and concentrated to 1.64 mg/ml. Three ul of S-ACE2 complex protein were applied to newly glow-discharged holy carbon film grids (Au R1.2/1.3, 300 meshes, Quantifoil, Germany). The grids were blotted with force 0 and blotting time of 3.0 s at 100% humidity and 4°C, followed by vitrification by plunge freezing into liquid ethane using Vitrobot Mark IV (Thermo Fisher Scientific, USA).

Dataset1 was collected on a Titan Krios G2 TEM (Thermo Fisher Scientific, USA) operating at 300 KV, equipped with a Gatan K2 Bioquantum direct detection camera (Gatan Company, USA) and an energy filter operating in zero-loss mode with a slit width of 20 eV. Dataset2 was collected on a Titan Krios G1 TEM (Thermo Fisher Scientific, USA) operating at 300 KV, equipped with a Gatan K3 camera (Gatan Company, USA). Images were recorded using SerialEM (version 3.8.4) with a beam-image shift method[2]. Each movie had an accumulated dose of 80 e /Å^2, fractionated into 50 frames (dataset1) or 40 frames (dataset2) with a pixel size of 1.36 Å (dataset1)

or 1.35 Å (dataset2). Images were collected at a range of defocus between -1.3 and -1.8 μm. A total of 5,401 movies were collected.

Image processing and model building

For cryo-EM image processing, all main steps were performed using Relion 3.1[3] or cryoSPARC 3.2[4]. Pyem[5] and UCSF Chimera[6] were used for format conversion of data files and reconstructions analysis, respectively.

In brief, 5,401 movie stacks were aligned by 5 × 5 patches with dose weighting and binned to 1.36 Å (dataset1) or 1.35 Å (dataset2) using Motioncor2[7]. The contrast transfer function parameters were estimated using GCTF[8]. 1,152,355 candidate particles were initially picked using Gautomatch without template (https://www2.mrc-lmb.cam.ac.uk/download/gautomatch-056/). Particles were extracted at bin4 level and followed by one round of 2D classification in Relion, giving a raw data set of 1,082,936 particles. Subsequentially, EMD-30661 map was low-pass-filtered to 40 Å and used as an initial model to perform 3D classification. 503,218 good particles were selected, and a map of 10.9 Å was generated. These particles were re-extracted at bin2 level, followed by a round of multi-reference 3D classification using a resolution-gradient reconstructed map. A set of 182,233 good-quality particles were selected, and then 3D classification was performed using resolution-gradient S-ACE2 maps and S maps as references. 64,541 S-ACE2 particles generated a map of 4.4 Å, after Bayesian polishing and auto-refine in Relion, and a map of 4.1 Å, after NU-Refinement in cryoSPARC. 95,436 S particles combined with 64,541 S-ACE2 particles yielded a reconstruction

with a resolution of 3.9 Å after Bayesian polishing and auto-refine in Relion, which was further increased to 3.7 Å after NU-Refinement in cryoSPARC. The resolution was estimated based on the gold-standard FSC using the 0.143 criterion. Local resolution was determined using cryoSPARC.

To build the atomic model of B.1.1.7 S and S-ACE2 model, the reported cryo-EM structure of B.1.1.7 S (PDB entry 7LWV) and S(N501Y)-ACE2 complex (PDB entry 7MJM) were used as initial models. We were able to trace most regions with side chains using Coot[9]. The model was further refined using PHENIX. The final model was validated using EMRinger[10]. All structure figures were prepared in UCSF ChimeraX[11] and Pymol (www.pymol.org).

Statistical analysis

Statistical analyses were performed with GraphPad Prism 5 software, using Student's unpaired two-tailed t-test and analysis of variance (ANOVA) test. P value less than 0.05 was regarded as significant differences: *P < 0.05; **P < 0.01; ***P <0.001.

References

1. Xia, S., *et al.* Potent MERS-CoV Fusion Inhibitory Peptides Identified from HR2 Domain in Spike Protein of Bat Coronavirus HKU4. *Viruses* **11**(2019).
2. Wu, C., Huang, X., Cheng, J., Zhu, D. & Zhang, X. High-quality, high-throughput cryo-electron microscopy data collection via beam tilt and astigmatism-free beam-image shift. *J Struct Biol* **208**, 107396 (2019).
3. Zivanov, J., Nakane, T. & Scheres, S.H.W. Estimation of high-order aberrations and anisotropic magnification from cryo-EM data sets in RELION-3.1. *IUCrJ* **7**, 253-267 (2020).
4. Punjani, A., Rubinstein, J.L., Fleet, D.J. & Brubaker, M.A. cryoSPARC: algorithms for rapid unsupervised cryo-EM structure determination. *Nat*

Methods **14**, 290-296 (2017).
5. Asarnow, D., *et al.* Structural insight into SARS-CoV-2 neutralizing antibodies and modulation of syncytia. *Cell* **184**, 3192-3204 e3116 (2021).
6. Pettersen, E.F., *et al.* UCSF Chimera--a visualization system for exploratory research and analysis. *J Comput Chem* **25**, 1605-1612 (2004).
7. Zheng, S.Q., *et al.* MotionCor2: anisotropic correction of beam-induced motion for improved cryo-electron microscopy. *Nat Methods* **14**, 331-332 (2017).
8. Zhang, K. Gctf: Real-time CTF determination and correction. *J Struct Biol* **193**, 1-12 (2016).
9. Emsley, P., Lohkamp, B., Scott, W.G. & Cowtan, K. Features and development of Coot. *Acta Crystallogr D Biol Crystallogr* **66**, 486-501 (2010).
10. Barad, B.A., *et al.* EMRinger: side chain-directed model and map validation for 3D cryo-electron microscopy. *Nat Methods* **12**, 943-946 (2015).
11. Pettersen, E.F., *et al.* UCSF ChimeraX: Structure visualization for researchers, educators, and developers. *Protein Sci* **30**, 70-82 (2021).

.

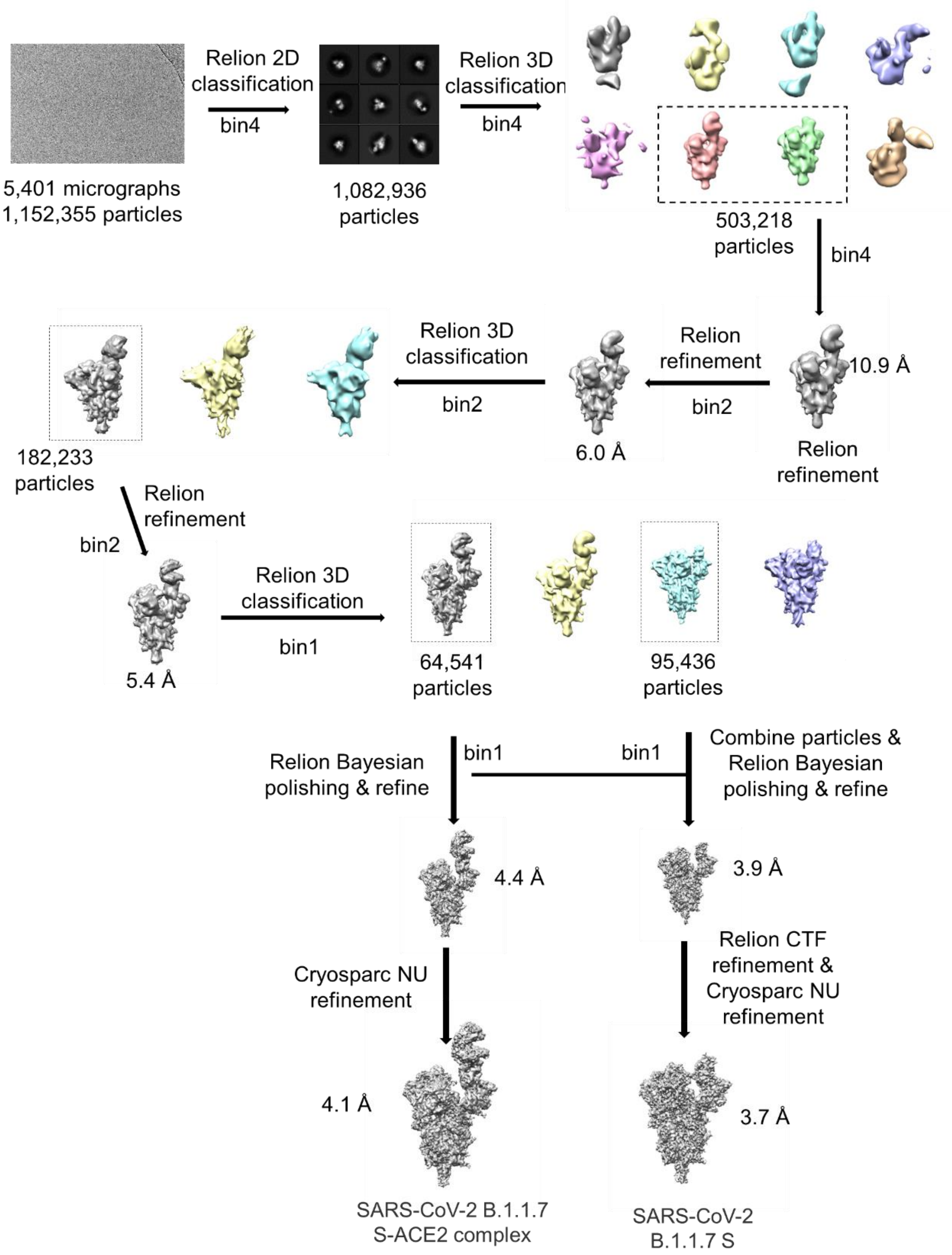

Supplementary Fig. S1. Workflow for cryo-EM structural determination of the SARS-CoV-2 B.1.1.7 S protein and its complex with hACE2.

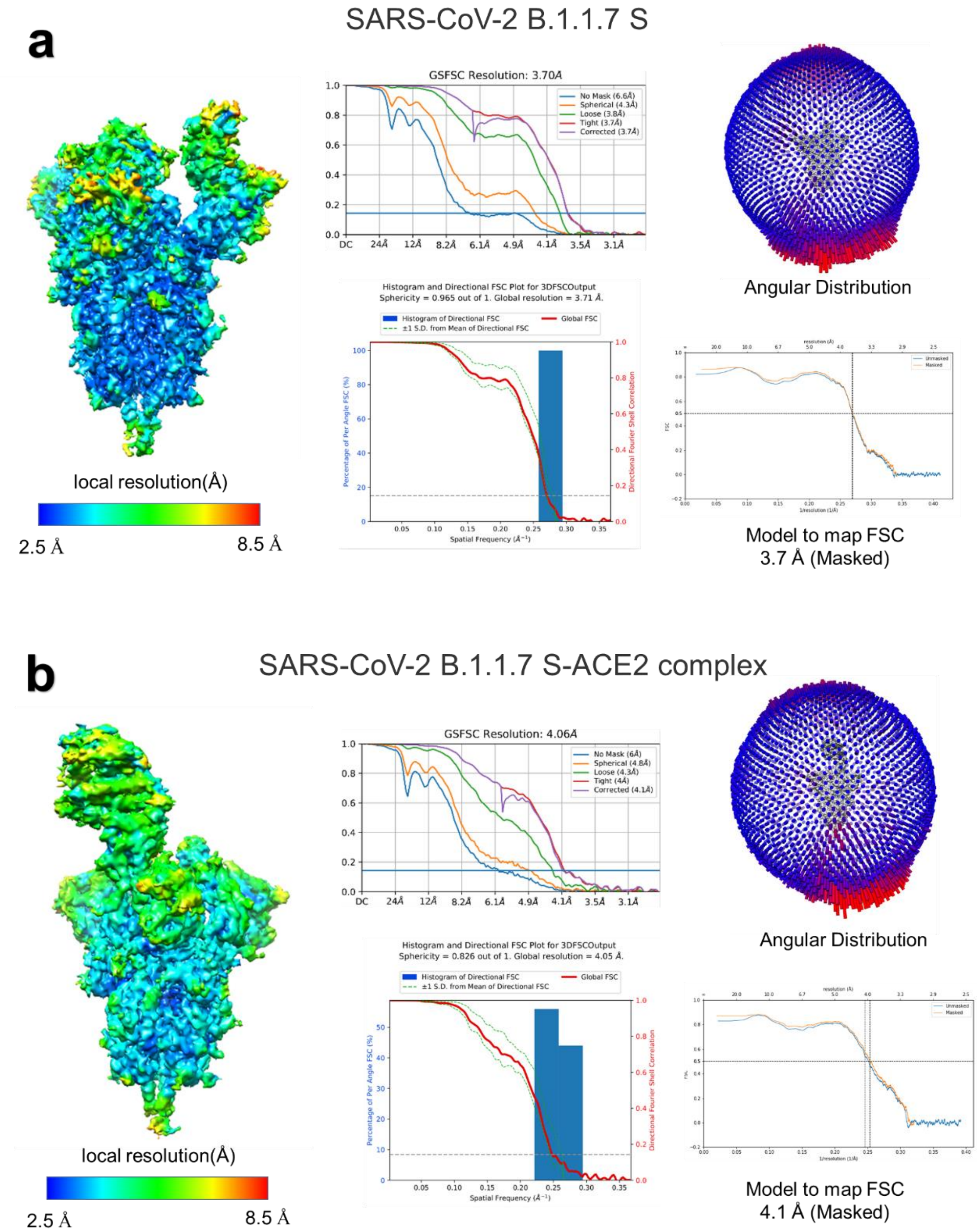

Supplementary Fig. S2. Map and model qualities for the cryo-EM structures of the SARS-CoV-2 B.1.1.7 S protein (a) and its complex with hACE2 (b).

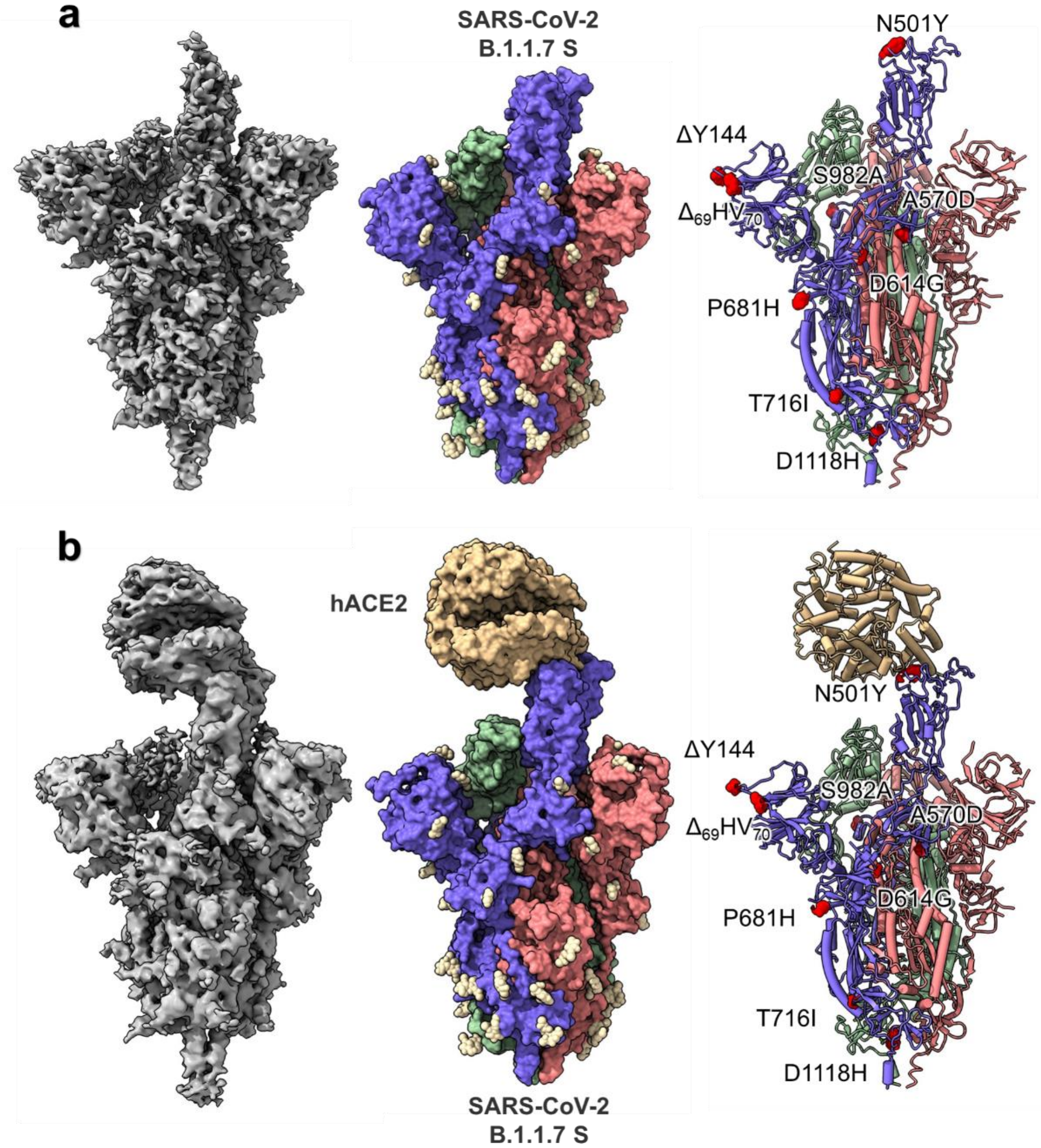

Supplementary Fig. S3. Overall structure of SARS-CoV-2 B.1.1.7 S (a) and the S-ACE2 complex (b).

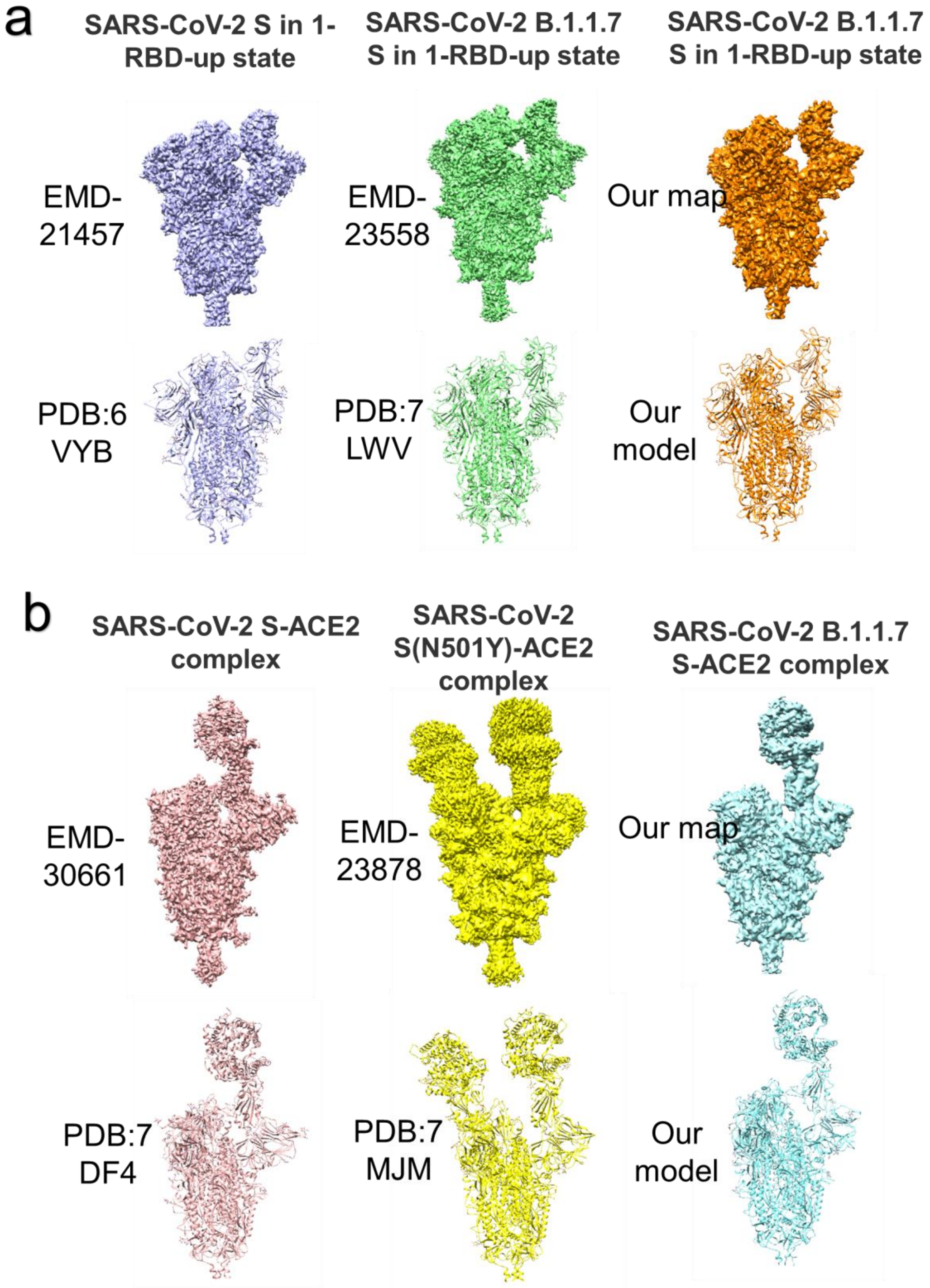

Supplementary Fig. S4. Structural comparison of different SARS-CoV-2 S proteins (a) and S-hACE2 complexes (b).

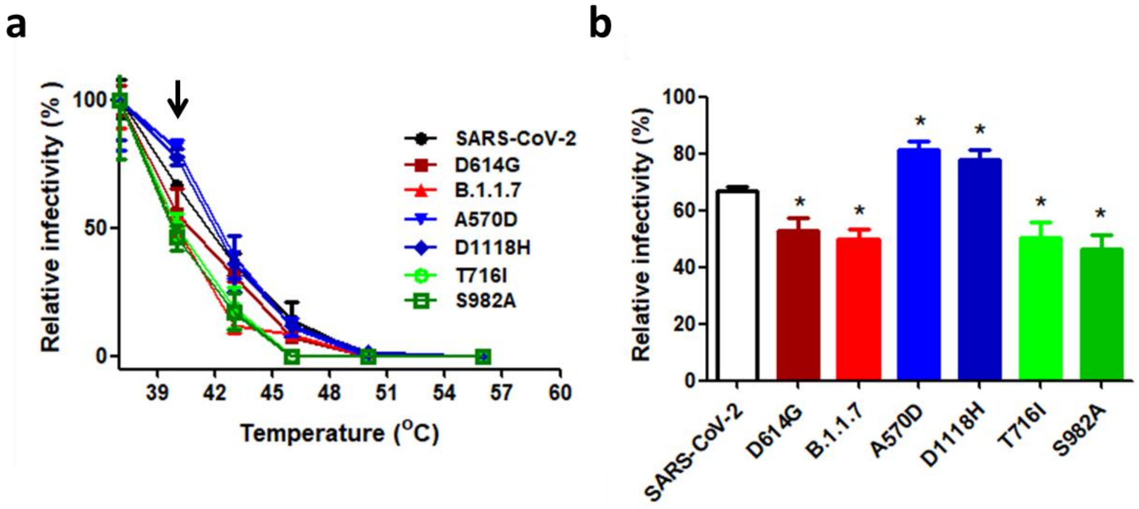

Supplementary Fig. S5. The thermostability assessment of SARS-CoV-2 or its variants S proteins. a. Pseudovirions were incubated at the indicated temperature (37 to 56 °C) for 2 h and then tested their relative infectivity at 37 °C. **b.** The relative infectivity of pseudovirions were incubated at 40 °C. As compared with the SARS-CoV-2 group, asterisks indicate significant differences (*P < 0.05).

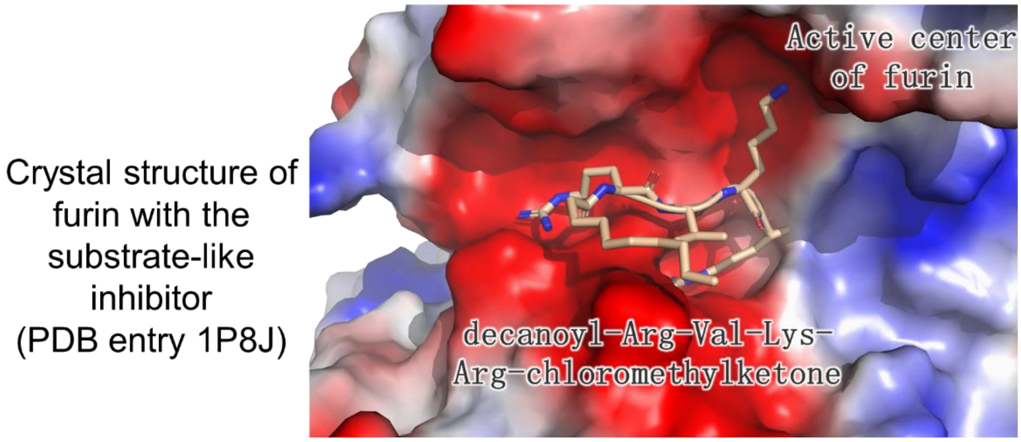

Supplementary Fig. S6. Structure of furin in complex with a substrate-like inhibitor.

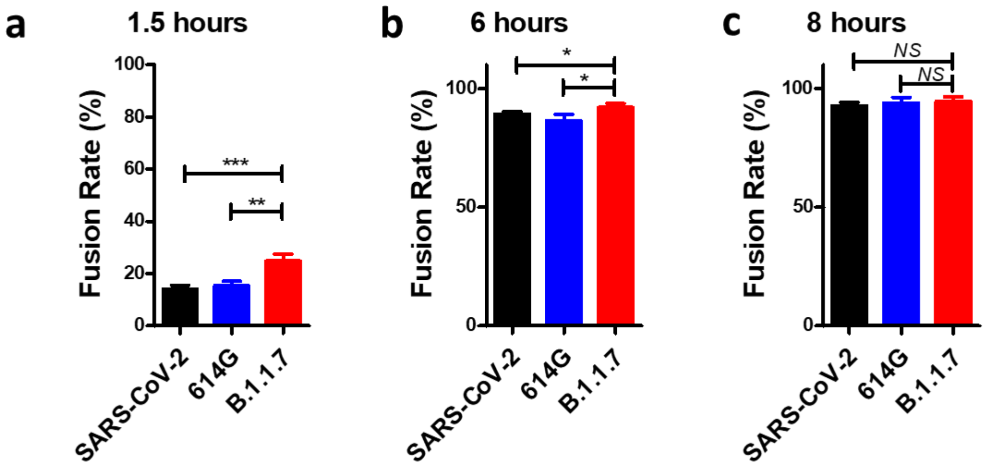

Supplementary Fig. S7. Fusion rate mediated by the WT, D614G and B.1.1.7 S proteins in Calu-3 cells after coculture for 1.5 (a), 6 (b) and 8 (c) hours. Asterisks indicate significant differences (*$P < 0.05$, **$P < 0.01$, ***$P < 0.001$); *NS*: no significance.

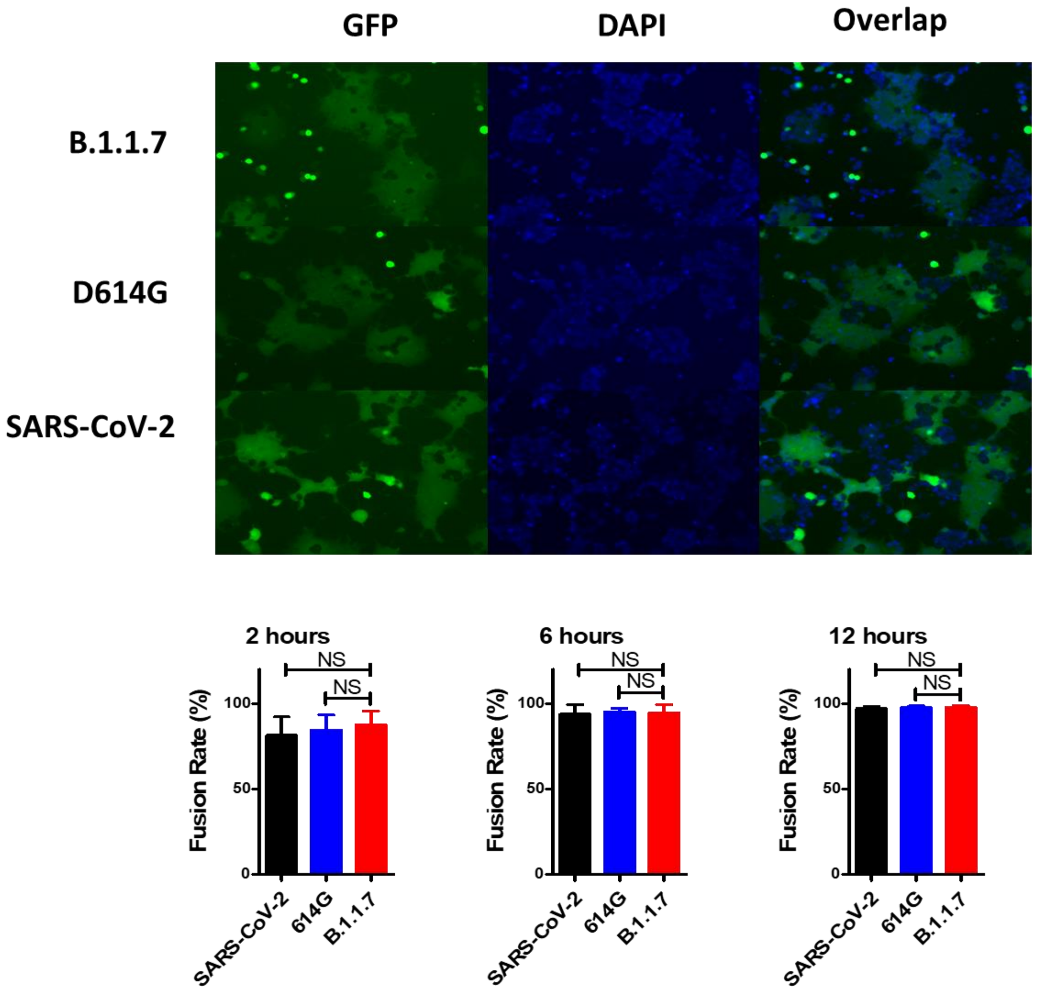

Supplementary Fig. S8. Fusion rate mediated by the WT, D614G and B.1.1.7 S proteins in 293T/ACE2 cells. “NS” indicate no significant difference.

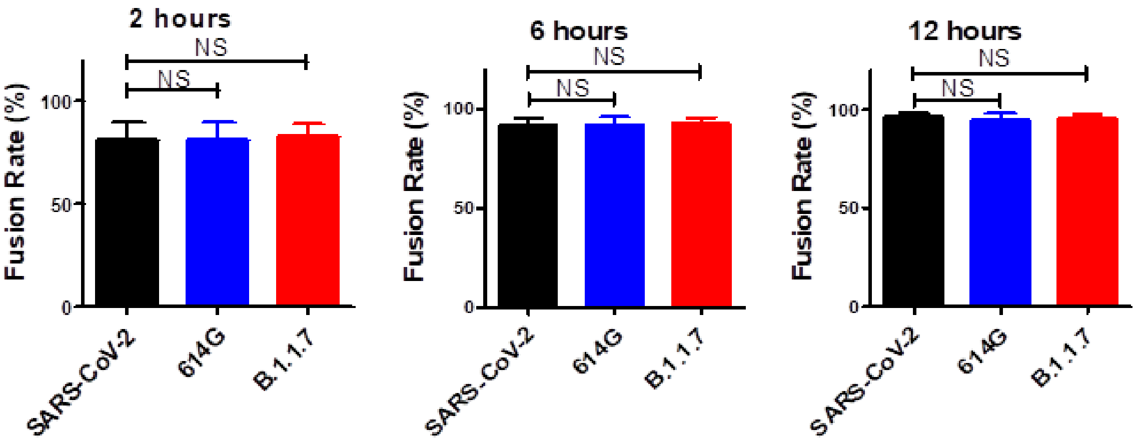

Supplementary Fig. S9. Fusion rate mediated by the WT, D614G and B.1.1.7 S proteins in 293T/ACE2/TMPRSS2 cells. “NS” indicate no significant difference.

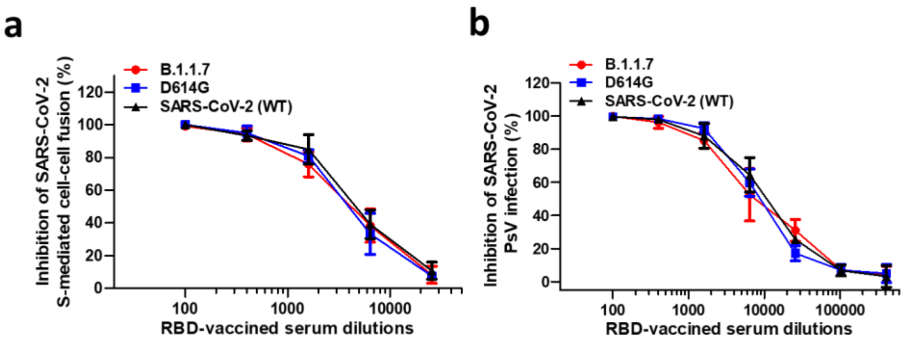

Supplementary Fig. S10. Inhibitory activity of serum from WT mice vaccinated with the SARS-CoV-2 RBD against WT, D614G and B.1.1.7 S-mediated cell–cell fusion (a) and pseudovirus infection (b).

Supplementary Table S1. Statistics of data collection, image processing and model building

**Structure**	**SARS-CoV-2 B.1.1.7 S in 1-RBD-up state**	**SARS-CoV-2 B.1.1.7 S with hACE2**
**PDB**	7FET	7FEM
**EMDB**	EMD-31563	EMD-31557
**Data Collection**		
	Dataset 1	Dataset 2
**Microscope**	Titan Krios G2	Titan Krios G1
**Voltage [kV]**	300	300
**Detector**	Gatan K2 Summit	Gatan K3 Summit
**Energy filter width [eV]**	20	/
**Automation software**	SerialEM	SerialEM
**Pixel size [Å/pixel]**	1.36	1.35
**Electron dose [e^-/Å^2]**	80	80
**No. of frames**	50	40
**Defocus range [µm]**	-1.3 ~ -1.8	-1.3 ~ -1.8
**Reconstruction**		
**Software**	cryoSPARC 3.2 and Relion 3.1	
**No. of particles**	159,977	64,541
**Symmetry**	C1	C1

**Map sharpening B-factor[Å^2]**	51.3	84.6
**Final resolution [Å]**	3.7	4.1
**Model Building and Refinement**		
**Building Software**	Coot	
**Refinement Software**	PHENIX	
**Rmsd (bond) [Å]**	0.006	0.013
**Rmsd (angle) [°]**	0.787	1.083
**MolProbity score**	2..37	2.38
**Model Composition**		
**No. of residues**	3003	3579
**Ligands**	None	
**Validation**		
**Ramachandran plot [%]**		
**Outliers**	0.07	0.14
**Allowed**	13.94	11.01
**Favored**	86.00	88.84
**Rotamer Outliers [%]**	0.08	0.00

Supplementary Video S1. Overall map of SARS-CoV-2 B.1.1.7 S in 1-RBD-up state with model fitted. Views are sliced to show the overall quality of the map and the fitted model.

Supplementary Video S2. Overall map of SARS-CoV-2 B.1.1.7 S-ACE2 complex with model fitted. Views are sliced to show the overall quality of the map and the fitted model.
